# Use of information communication technologies by older people and telemedicine adoption during COVID-19: a longitudinal study

**DOI:** 10.1093/jamia/ocad165

**Published:** 2023-08-12

**Authors:** Likun Mao, Gretta Mohan, Charles Normand

**Affiliations:** Department of Economics, University of Aberdeen, King’s College, Aberdeen AB24 3FX, United Kingdom; The Irish Longitudinal Study on Ageing (TILDA), Trinity College Dublin, Trinity Central, Dublin D02 R590, Republic of Ireland; Economic and Social Research Institute, Dublin 2 D02 K138, Republic of Ireland; Centre for Health Policy and Management, Trinity College Dublin, Dublin 2, Republic of Ireland; Cicely Saunders Institute of Palliative Care, Policy & Rehabilitation, Kings College London, London SE5 9PJ, United Kingdom

**Keywords:** older population, Ireland, telemedicine, healthcare, COVID-19

## Abstract

**Objectives:**

To investigate how information communication technology (ICT) factors relate to the use of telemedicine by older people in Ireland during the pandemic in 2020. Furthermore, the paper tested whether the supply of primary care, measured by General Practitioner’s (GP) accessibility, influenced people’s telemedicine options.

**Method:**

Based on 2 waves from The Irish Longitudinal Study on Ageing, a nationally representative sample, multivariate logistic models were applied to examine the association between pre-pandemic use of ICTs and telemedicine usage (GP, pharmacist, hospital doctor), controlling for a series of demographic, health, and socioeconomic characteristics.

**Results:**

Previously reported having Internet access was a statistically positive predictor for telemedicine usage. The availability of high-speed broadband Internet did not exhibit a statistical association. The association was more prominent among those under 70 years old and non-Dublin urban areas. People with more chronic conditions, poorer mental health, and private health insurance had higher odds of using telemedicine during the period of study. No clear pattern between telemedicine use and differential geographic access to GP was found.

**Discussion:**

The important role of ICT access and frequent engagement with the Internet in encouraging telemedicine usage among older adults was evidenced.

**Conclusion:**

Internet access was a strong predictor for telemedicine usage. We found no evidence of a substitution or complementary relationship between telemedicine and in-person primary care access.

## Introduction

As the use of information communication technologies (ICTs) has penetrated more aspects of modern life, some policymakers have become concerned that older adults are at a greater risk of digital exclusion.^1^ ICT has provided additional options for the delivery of healthcare services, where patients may attend appointments and receive care from their own homes. Such alternatives emerged as a vital channel for the provision of continued healthcare during the coronavirus disease 2019 (COVID-19) pandemic crisis and the associated government-mandated lockdowns. Correspondingly, the deployment of telemedicine has been receiving increasing attention among researchers and policymakers.^2–9^

An established line of research on telemedicine has investigated how people use ICT to access healthcare, and what individual- or institutional-level characteristics determine eHealth-related behaviors.^7^^,^^10–12^ Technology-related factors such as digital literacy (both patients and clinicians) and ICT infrastructure have been widely documented as important barriers to delivering remote health services.^7^^,^^10^^,^^11^^,^^13^ These factors may be of particular relevance to older populations who may have comparatively lower ICT access, more technostress experiences^14^ and additional learning requirements for engaging with eHealth.^15^ Research suggests an influence of digital connectivity, attitudes to technologies, relevant ICT experience and literacy on older adults’ use of health-related technology.^7^^,^^16^^,^^17^

It is informative and important to provide updated evidence to advance the understanding of the evolving role played by telemedicine in healthcare. In the context of the pandemic, emerging studies examined patients’ characteristics and telemedicine usage based on large samples.^5^^,^^3^^,^^6^^,^^18–20^ Some are descriptive, primarily investigating the upsurge in telemedicine consultations before and after the pandemic using cross-sectional data.^5^^,^^18–20^ When it comes to quantifying the statistical relationship, Chunara et al. explored health record data of patients at New York University Langone Health consisting of over 8000 healthcare providers. After controlling for individual- and community-level factors, results from multilevel regressions showed racial and ethnic disparities in telemedicine access; black people living in lower socioeconomic status (SES) neighborhoods were less likely to access care through telemedicine compared to white people. Huang et al. examined the role of sociodemographic characteristics, technological access, and patients’ own personal primary care provider in choosing a telephone or video visit. As suggested, positive predictors of choosing a video visit versus a telephone visit are younger age (under 18) and older age (over 65), white and Asian race, residence in a high SES area, and technological access. Generally, these cross-sectional studies are based on all-age adult patients’ data provided by relevant and localized healthcare providers including limited information about individual-level characteristics.

Following this background, the current study used microdata from 2 waves of The Irish Longitudinal Study on Ageing (TILDA), a nationally representative dataset on adults over 50 in Ireland, to examine the longitudinal association between ICT factors and telemedicine usage among the older population. It aimed to provide insight as to whether previous engagement by older people with digital technologies influences the propensity to use telemedicine. Furthermore, using available geographic information on the provision of GPs, we considered the potential effect of the supply of primary care in an older person’s area.

The analysis of these factors was conceptually informed by The Technology Acceptance Model (TAM) which had been widely used in studying influential factors in people’s potential acceptance or rejection of technologies.^21–24^ Four components are proposed: performance expectancy (PE), effort expectancy (EE), social influence (SI), and facilitating conditions (FC). Specific to our analysis, ICT access was examined in relation to FC, the infrastructure aspect. PE and EE correspond to people’s perceived benefits and ease of use, which could be reflected by people’s ICT-related activities in some ways. GP supply is an important enabling resource in the use of healthcare.^25^^,^^26^ A lack of supply of GPs in one’s area may prompt greater adoption of remote health services that help overcome physical and proximal access barriers, which was linked to the PE aspect.

This work adds new insights to the current literature in 3 aspects. First, to our knowledge, this is the first multivariate analysis of telemedicine based on nationally representative individual-level data among emerging studies of the pandemic context, and it is also the first empirical analysis based on a European sample. (So far, only a few studies are based on European samples before the pandemic.^11^^,^^27–30^) Second, we focused on the role of technological factors that were suggested as important determinants of telemedicine usage.^7^ Third, we utilized the unique geographical information about the supply of GPs^31^ to control for the availability of GPs in an older person’s locality, where a poorer level of access to GPs in an older person’s locality may prompt older people to make online healthcare appointments. This allowed us to explore whether telemedicine options may help mitigate regional disparities in the accessibility of primary healthcare resources. Research suggests that geographical factors affect access to general practice care and community care.^32^^,^^33^ As Ireland has a large rural population, telemedicine has the potential of improving rural health service delivery.^34^^,^^35^

## Data and sample

This study used information from TILDA that collects data on the health, economic and social circumstances of community-dwelling adults, aged 50 years and over, in Ireland. TILDA has a two-stage sampling design.^36^ The survey combines 2 main forms: Computer-Assisted Personal Interview and Self-Completion-Questionnaires (SCQs). The first wave covers the period from October 2009 to February 2011 with 8504 participants, and the follow-up waves take place approximately every 2 years. The analytical sample was restricted to the people who participated in the fifth wave (collected in 2018) and a special COVID-19 wave (collected from June 2020 to November 2020) where participants of both waves resided at the same address since wave 5. Longitudinal weights were used to adjust for sample attrition and sampling design. With complete information for the key variables of research interest, the analytical sample consisted of 2607 individuals (from 2045 households), among which 1115 were male (42.8%) and 1490 were female (57.2%).

Table 1 presents descriptive statistics of the sample as reported in wave 5. The average age was 68.9 years old, and 73.5% were married and living together. 26.6% of the sampled participants lived in the capital city, Dublin, while 27.9% were in other cities or towns, and the remaining were in rural areas. The average household size was 2.2 persons. The average household gross assets were 556 thousand euros, and the majority were living in their own house (94.2%). More than half of the respondents were retired. The majority of the sample had no disability (92.1%). Over half reported at least a very good level of general health or mental health.

**Table 1.** Summary statistics.

	All		Male	Female
	(*N* = 2607)		(*N* = 1115)	(*N* = 1490)
	**Mean**	**SD**	**Mean**	**Mean**
Age	68.9	7.9	69.9	68.2
Household size	2.2	1.0	2.3	2.2
Household gross assets (in thousands)	556.39	680.12	599.08	523.88
	**Proportion**	**SD**	**Proportion**	**Proportion**
Has own house	0.942	0.234	0.954	0.932
Dublin	0.266	0.442	0.274	0.260
Rural area	0.455	0.498	0.446	0.462
Married	0.735	0.441	0.799	0.687
Education: third/higher	0.444	0.497	0.402	0.475
Employed/self-employed	0.298	0.458	0.345	0.263
Retired	0.527	0.499	0.601	0.473
Chronic condition: no	0.074	0.262	0.082	0.068
Chronic condition: 1-2	0.480	0.500	0.503	0.462
Chronic condition: ≥3	0.358	0.480	0.303	0.399
Self-rated health: excellent	0.153	0.360	0.140	0.164
Self-rated health: fair/poor	0.114	0.318	0.126	0.105
Disability: no	0.921	0.270	0.920	0.922
Mental health: excellent	0.182	0.386	0.175	0.187
Mental health: fair/poor	0.071	0.257	0.064	0.077

### Telemedicine measures

**Figure 1.**
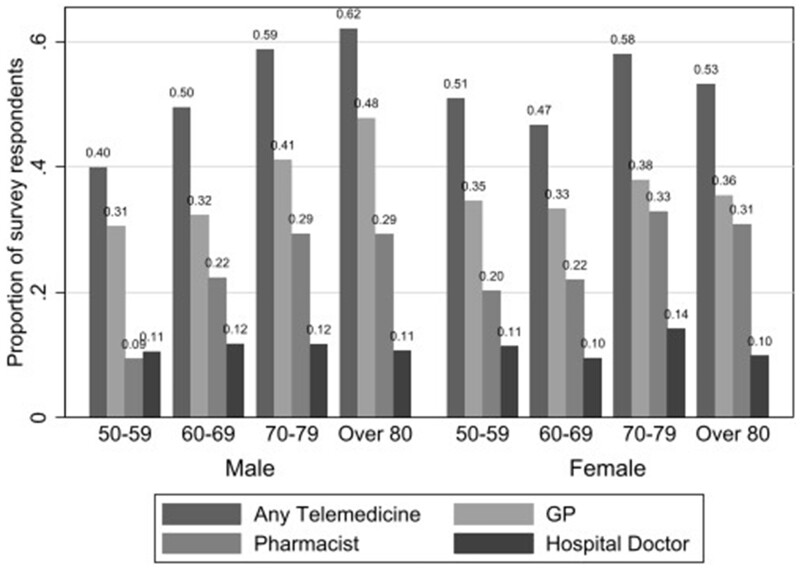
Telemedicine usage by gender and age group.

Variables about the use of telemedicine in 2020 are contained in TILDA’s COVID-19 study in the form of a SCQ. Respondents were asked, whether they “avail of a telephone or online appointment from any of the following health services: GP, pharmacist, hospital doctor and any other health professional.” That is, whether the healthcare-related appointment was delivered by a GP, a pharmacist or a hospital doctor, or other health professional. Where a respondent indicated they utilized a remote appointment with a particular clinician, a binary variable relating to that particular healthcare service was coded as one. If none of the 4 options was reported, these cases were categorized as non-user. More than half the sample (52.5%) reported the use of telemedicine, either GP, pharmacist, hospital doctor, or other (for more details see Table S1). The most prevalent, in order, are GP (36.1%), pharmacist (25.5%), and hospital doctor (11.4%). About one-third reported only one category among the 3. There were also variations of telemedicine use across gender and age groups (Figure 1). A small difference in the prevalence of telemedicine by gender was observed for the sample, although women are often considered to be more active in eHealth (eg, Refs^27^^,^^37^). Notably, the proportion of telemedicine users was generally stable over different ages for women. By comparison, there was an increasing tendency to use telemedicine by age among men, particularly for the pharmacist: the figure increased from 9% in the male group under 70 years old to 29% among males over 70 years old.

### ICT variables

In the SCQ of wave 5, participants were asked to report their Internet access and related behaviors such as which device they used to access the Internet, frequency of use of the Internet, and recent online activities. (Original SCQ questionnaire questions are provided in the Supplementary file.) As presented in Table S1, the majority reported having Internet access (86.4%) either at home or in other places (e.g., library). The most common devices used to access the Internet were a smartphone or tablet (75.7%). Desktop computers and laptops (70.5%) ranked second. In general, women were slightly more active Internet users, particularly for social aspects such as telephone/video calls and social media. By age groups, internet use was less common in older groups. Over 90% of those under 70 had Internet access, and over 70% used the Internet every day. For the 70 to 79 group, about half reported a daily frequency, and the figure drops to about 30% on average for people over 80.

The measure of high-speed broadband availability was created by spatially linking each residence of a TILDA participant with Ireland’s National Broadband Plan (The National Broadband Plan defines high-speed broadband as having a download speed of 30 megabits per second (Mbps) or higher) map for 2017.^38^ Areas where high-speed broadband was available could be identified. Although this proxy did not directly measure whether a respondent has taken up high-speed broadband, it provided an indication of the likely connectivity available at the residence. 71.7% had high-speed broadband available. The figure was 97.2% for TILDA residents in the Dublin area, and 89.2% in other cities or towns, though the coverage was only 46.0% in rural areas.

### Statistical method

To examine the longitudinal association between previous reports of older people’s ICT access and usage in 2018 and their adoption of telephone/online health services in 2020, we employed a multivariate logistic model specified as follows:


$$\mathrm{Pr}\left(Y_{it}=1|{\mathrm{ICT}}_{it-1},X_{it-1},\;\varepsilon_{it}\right)=F(\alpha {{\mathrm{ICT}}_{it-1}+\beta X}_{it-1}+\varepsilon_{it})$$

Where $Y_{it}$ is a binary outcome variable indicating whether an older person used a telemedicine service in 2020. $X_{it-1}\;$is a vector of covariates taken from time *t*−1, representing TILDA wave 5 (2018), including our controls for a range of demographic, socioeconomic, and health characteristics that are likely to affect both telemedicine use and ICT variables: age, age-squared (divided by 100), gender, residence location (Dublin, other cities, or rural area), marital status (married, never married, separated/divorced, widowed), whether born in Ireland, childhood socioeconomic condition (excellent, average, poor), childhood health condition (excellent, very good, good, fair/poor), education level (primary/none, secondary, third/higher), household size, current labor market status (retired, employed/self-employed, not working, never worked), household assets, house ownership, healthcare entitlements (only public health insurance, only private health insurance, dual covered), and health conditions: chronic disease (none, 1–2, or 3+), disability (no, IADL only, any ADL), self-rated mental health condition (excellent, very good, good, fair/poor), self-rated general health condition (excellent, very good, good, fair/poor). ${\mathrm{ICT}}_{it-1}$ denotes a range of Internet-related variables recorded for wave 5. A Principal Component Analysis (PCA) was also undertaken to construct a proxy of a general Internet activity level based on the ICT variables available. (PCA was implemented in STATA 16 using a direct command. The general activity index is a weighted sum of 8 Internet activity variables. The identified weights for imputation are relatively even across all activities. More details are available upon request.) $\varepsilon_{it}$ is the error term. Regressions were separately repeated over each outcome variable and each ICT variable of interest. All regressions included time fixed effects for the interview month to control for the changing pandemic situations. In addition to analyzing the whole analytical sample, separate analyses were carried out for subgroups by gender, those under 70 years and those 70 years and over, as well as for those by urban/rural locations.

α is the parameter of interest reflecting the association conditioning on a set of controls. The rich information from TILDA allowed us to control for confounding variables that could affect the outcome variables, and to examine the associations between other individual characteristics and telemedicine adoption in a broader sense. We performed several sensitivity checks: the first was model specification experimenting with an alternative set of controls. Second, we trimmed the sample by excluding some cases that have the potential to affect the reliability of our estimates. We excluded heavy healthcare users, specifically those in the top 10% in terms of GP visits in wave 5. We also excluded cases of people who indicated that they were extraordinarily concerned about the uncertainties of the COVID-19 situation, who may potentially seek more remote consultations pertinent to COVID-related health issues. (In the original SCQ questionnaire, respondents were asked “Overall, on a scale from 1 to 10, how concerned are you about the COVID-19 pandemic.” We excluded the cases of the most concerned group reporting the highest score. The question was designed to capture people’s general sense of the uncertain impact of the pandemic on some broader issues such as the economy and health.^39^ We regarded this only as a proxy for one’s concern over individual health issues.) In addition, we separated samples by those who reported they had experienced any healthcare service delay during COVID—since it could be the case that the estimates were largely driven by the people looking for remote health services as temporary substitutes. Third, we stratified our sample by educational level to test the possibility that estimates could be driven by people from a high socioeconomic background who are likely to be positively associated with ICT resources, health resources and health literacy or lifestyles. Relevant results are provided in Tables S2 to S4 and demonstrated the robustness of our main findings.

To analyze the potential association between telemedicine and in-person health services, we considered the potential heterogeneity in telemedicine usage by GP accessibility in Ireland. Building upon geographical information about the availability of GPs in the vicinity of each household, 3 measures were used to characterize the local supply of GP services: road network distance (kilometers) from TILDA respondent’s residence to the nearest GP, the number of residential addresses within a proxy geographical catchment area of the nearest GP, and the total number of GPs within walking distance (1.6-km radius) of a respondent’s residence.^31^ Respectively, these variables enabled us to characterize geographical accessibility, local GP capacity, and the choice of GPs available for each respondent. (Under a special research access agreement, the home address of TILDA participants were mapped and linked to information on the location of GPs across Ireland [XY coordinates]. More details are provided in the Supplementary files.) Taking account of this regional disparity and the skewness of original variables (the data on GP accessibility exhibited apparent urban-rural differences: rural residents have considerably greater distances to GPs [7203 m on average, compared to 461 m in Dublin] and fewer GPs available within a walking distance [a 20-min walk]. However, the proxy for capacity of the local GP did not vary considerably), we constructed tertiles of each accessibility measure within a general location (Dublin, other cities, and rural areas), and these 3 quantile dummies (lower 33%, 33%–66%, top 33%), and interaction terms with ICT variables were included in our main estimation equation to investigate the potential differential effect of Internet access. For the measure of the number of total GPs, the quantile groups were assigned reversely, so for all 3 measures, the higher percentiles mean less geographic GP accessibility.

## Results

### ICT and telemedicine

Our multivariate analysis results are presented in Table 2. In panel A, statistically significant and positive coefficients of Internet access were estimated in predicting the use of telemedicine: on average, older people with Internet access had a 1.6 higher odds of using a healthcare service on a remote basis than those who did not have Internet access. This was largely driven by remote appointments with a GP or pharmacist. The association between Internet access and the use of remote consultations with hospital doctors was not statistically significant. The gender difference was not substantial, though males had a slightly higher tendency to use remote healthcare (53.8% vs. 51.5%). As to age-related heterogeneities, results in columns 4 and 5 showed a stronger association among those under the age of 70. The odds ratios reached as high as 3, and all were statistically significant at the 1% level. By comparison, for the older group (70 years and above), the association was weak and statistically insignificant. Additional subgroup analyses demonstrated a higher estimated odds ratio of telemedicine among urban dwellers outside Dublin, than for those residing in the capital city (Table S4).

**Table 2.** Association between the use of telemedicine services in 2020 and Internet access, high-speed broadband availability in 2018.

Telemedicine service	(1)	(2)	(3)	(4)	(5)
	All	Male	Female	Age < 70	Age ≥ 70
Panel A: Internet access in wave 5					
Any health services	1.587***	1.691**	1.598**	3.094***	1.159
	(0.264)	(0.421)	(0.356)	(0.971)	(0.224)
GP	1.622***	1.659*	1.665**	2.911***	1.216
	(0.279)	(0.430)	(0.381)	(0.968)	(0.245)
Pharmacist	1.676***	1.615*	1.668**	3.249***	1.270
	(0.316)	(0.416)	(0.416)	(1.353)	(0.273)
Hospital doctor	1.192	1.355	1.120	2.149	0.934
	(0.298)	(0.479)	(0.388)	(1.118)	(0.270)
N	2607	1115	1492	1449	1153
Panel B: High-speed broadband availability					
Any health services	0.991	0.927	1.025	1.127	0.781
	(0.134)	(0.185)	(0.184)	(0.196)	(0.167)
GP	1.118	1.052	1.132	1.258	0.881
	(0.161)	(0.214)	(0.221)	(0.235)	(0.199)
Pharmacist	0.846	0.688	0.856	0.798	0.969
	(0.135)	(0.157)	(0.182)	(0.172)	(0.231)
Hospital doctor	0.767	1.110	0.508**	0.667	0.915
	(0.180)	(0.367)	(0.171)	(0.222)	(0.320)
N	2571	1099	1472	1427	1139

Standard errors in parentheses were clustered at the household level. Odds ratios reported. Any health services means availing of any remote appointment with a GP, pharmacist, or hospital doctor.

* *P* < 0.1,

** *P* < 0.05,

*** *P* < 0.01.

No statistically significant association between high-speed Internet availability at a TILDA respondent’s residence and engagement with telemedicine was reported in panel B. Despite the insignificant overall effect, there was some indication of regional heterogeneity (Table S4): the signs of a lower likelihood of using telemedicine among other cities were marginally significant in some cases, whereas the association appeared to be the opposite for Dublin residents.

Next, we moved on from general ICT access to examine various ICT-related behaviors, which could help with further identifying the relationship between telemedicine adoption and underlying technology-related factors from an individual perspective. Results are summarized in Table 3. The first 2 columns indicate that older people who used the internet daily in 2018 had a 1.3 greater odds of availing of telemedicine in 2020 than those who never used the internet. The estimates indicated that this association was predominantly driven by males, although women reported more Internet use in general. A positive association was estimated between telemedicine and using computers to access the Internet rather than other mobile devices such as smartphones and tablets. Again, the association was stronger and only statistically significant among men in most cases, except for remote appointments with hospital doctors. Compared to no previous engagement with online activities, those who reported previous use of emails and searching for information were estimated to have a 1.3 higher odds of undertaking remote GP appointments. No clear associations for other Internet behaviors were uncovered. (Results are available upon request.) The general ICT activity score showed no strong association with telemedicine except for some positive signs in the case of using a GP on a remote basis.

**Table 3.** Multivariate estimation results: association between use of telemedicine in 2020 and Internet activities reported in 2018.

Telemedicine service	Any health service			GP			Pharmacist			Hospital doctor		
	(1)	(2)	(3)	(4)	(5)	(6)	(7)	(8)	(9)	(10)	(11)	(12)
	All	Male	Female	All	Male	Female	All	Male	Female	All	Male	Female
Daily Internet users	1.314**	1.939***	0.997	1.393**	1.778***	1.102	1.325*	1.767***	1.061	0.961	0.938	1.062
	(0.169)	(0.360)	(0.175)	(0.184)	(0.332)	(0.202)	(0.196)	(0.373)	(0.218)	(0.193)	(0.258)	(0.307)
Use personal computer	1.401***	1.732***	1.278	1.501***	1.871***	1.321	1.175	1.305	1.084	1.647**	1.287	2.196***
to access Internet	(0.179)	(0.331)	(0.217)	(0.196)	(0.376)	(0.226)	(0.173)	(0.285)	(0.214)	(0.341)	(0.351)	(0.663)
Sending/receiving emails	1.074	1.299	0.998	1.350**	1.501**	1.298	1.195	0.976	1.464*	0.946	1.392	0.779
	(0.135)	(0.237)	(0.174)	(0.177)	(0.279)	(0.242)	(0.173)	(0.218)	(0.292)	(0.188)	(0.374)	(0.213)
Telephone/video call	0.914	1.103	0.775*	0.991	1.029	0.909	0.891	0.955	0.842	1.091	1.729**	0.846
(via webcam)	(0.106)	(0.181)	(0.119)	(0.120)	(0.171)	(0.147)	(0.117)	(0.182)	(0.150)	(0.199)	(0.456)	(0.220)
Information search for learning, research	1.182	1.272	1.157	1.313**	1.342	1.262	1.185	1.415	1.027	0.997	0.835	1.189
or finding facts	(0.156)	(0.240)	(0.210)	(0.181)	(0.263)	(0.239)	(0.190)	(0.314)	(0.220)	(0.203)	(0.235)	(0.350)
ICT activity score	1.041	1.090*	1.010	1.080**	1.093*	1.061	1.054	1.032	1.076	1.016	1.092	0.977
	(0.037)	(0.056)	(0.049)	(0.040)	(0.056)	(0.054)	(0.043)	(0.061)	(0.060)	(0.059)	(0.084)	(0.084)
N	2607	1115	1492	2607	1115	1492	2607	1115	1492	2607	1115	1492

Standard errors in parentheses were clustered at the household level. All estimates were adjusted in the same way as Table 2. For all other ICT-related variables, the reference category was the group who did not report the specified ICT activity.

Statistical significance indicated by

* *P* < 0.1,

** *P* < 0.05,

*** *P* < 0.01.

### Sociodemographic factors and telemedicine

There were statistically significant coefficients on some other controls (Table S5). First, most noticeably, statistically significant associations emerged for some specific health-related characteristics. More chronic conditions were strongly associated with the adoption of telemedicine, mostly driven by cardiovascular conditions. (Full results are available upon request. We further separated the number of cardiovascular diseases [e.g., angina, heart attack, heart failure, diabetes, stroke, high cholesterol], and other chronic conditions [e.g., asthma, lung disease, arthritis, cancer, osteoporosis, Parkinson’s, cataracts].) Poor mental health was estimated as a strong predictor of telemedicine use, with a 1.7 higher odds compared to good mental health. In terms of the general level of health, compared to good self-rated health, fair/poor health was estimated to have a marginally significant higher odds of telemedicine usage. Reporting a disability was not associated with remote healthcare consultation. Second, the estimates revealed little predictive power of socioeconomic characteristics such as educational level and household assets in explaining the variation in using telemedicine. Third, the estimates pointed to regional differences: rural residents had lower odds of using remote appointments for GP and hospital doctors. In general, a greater tendency to adopt telemedicine was observed for urban areas outside Dublin, and substantially higher odds of using remote access to pharmacists were estimated for areas outside Dublin including rural areas. Fourth, while many demographic variables were statistically insignificant, lower odds of telemedicine use were found for those born outside of Ireland. Finally, those covered by only private insurance had significantly lower odds of using telemedicine, except for pharmacists.

### GP accessibility and healthcare

Table 4 reports the associations between telemedicine usage and geographic GP accessibility, as well as the heterogeneous association between ICT and telemedicine by the level of GP accessibility. For the majority of the indicators of geographic GP accessibility, a lack of a statistically significant influence was demonstrated. There were some signs of a greater tendency to use telemedicine in the group with a mid-level of GP work capacity (i.e., the second tertile represented by the variable 33%–66% catchment). An estimated odds ratio of approximately 2, indicates that a person with a mid-level of GP capacity in their area had twice the odds of using remote GP services compared to the baseline group who benefit from the highest level of GP capacity. Regarding ICT, with an exception for the case of telemedicine for a GP consultation, the coefficients on the interaction terms between internet access and GP accessibility were higher and above one in the group featuring the longest distance (top 33%) to the nearest GP (columns [1] and [7]). The results suggest a greater association between internet access and the use of digital healthcare as GP accessibility decreases. However, there was no consistent pattern for other types of telemedicine, and no statistically significant results suggesting a heterogeneous impact on telemedicine use by GP accessibility.

**Table 4.** Heterogenous association between use of telemedicine in 2020 and indicators of geographical accessibility of GPs, and the interaction of GP accessibility with internet access in 2018.

Telemedicine type	Any health service			GP			Pharmacist		
	(1)	(2)	(3)	(4)	(5)	(6)	(7)	(8)	(9)
	Distance	Number	Catchment	Distance	Number	Catchment	Distance	Number	Catchment
33%–66% percentile	1.077	0.469*	1.352	1.494	0.461	2.006**	0.934	0.670	1.231
(ref: lowest 33% percentile)	(0.336)	(0.214)	(0.443)	(0.496)	(0.238)	(0.705)	(0.339)	(0.387)	(0.481)
Top 33% percentile	0.888	0.936	0.986	1.001	1.007	1.179	0.946	1.256	1.065
	(0.292)	(0.294)	(0.314)	(0.356)	(0.329)	(0.406)	(0.368)	(0.452)	(0.400)
33%–66% × internet access	0.730	1.846	0.771	0.553*	1.677	0.577	0.849	1.728	0.719
(ref: lowest 33% × internet access)	(0.249)	(0.899)	(0.273)	(0.199)	(0.904)	(0.218)	(0.338)	(1.060)	(0.304)
Top 33% × internet access	1.283	1.071	1.154	0.942	0.873	0.901	1.270	0.831	0.946
	(0.455)	(0.360)	(0.402)	(0.360)	(0.304)	(0.337)	(0.532)	(0.320)	(0.388)
N	2567	2571	2571	2567	2571	2571	2567	2571	2571

Standard errors in parentheses were clustered at the household level. Odds ratios reported. For each characteristic of GP accessibility, three quartile groups were constructed within general residence locations by Dublin, other cities/towns, and rural areas. Higher quartile groups reflect less GP accessibility.

* *P* < 0.1,

** *P* < 0.05,

*** *P* < 0.01.

## Discussion and conclusion

Research has documented how telemedicine can enable patients to access more flexible healthcare services through wider online platforms.^7^^,^^9^ By necessity, the COVID-19 pandemic has also expanded healthcare practitioner’s and the public’s adoption of, and experiences with, telemedicine channels.^2^ Contributing to the ongoing discussion, this study examined factors affecting telemedicine use by older people. A longitudinal analysis focused on the patient side, demonstrating a statistically significant positive association between previous Internet access and telemedicine usage, controlling for a series of demographic, health, and SES characteristics. However, the use of remote clinician appointments was not statistically significantly affected by whether the older person’s residence had high-speed broadband Internet available. The general level of ICT activity and people’s specific ICT behavioral patterns did not show a strong statistical correlation with older people’s use of telemedicine.

The association between ICT factors and telemedicine use was pronounced particularly for GPs and pharmacists, which have greater accessibility in communities. In Ireland, as in many other countries, GPs are the most common point of contact in primary care, acting as gatekeepers to secondary healthcare services such as hospital care. Pharmacists also provide a key role in community healthcare provision, dispensing medication and healthcare advice, typically in a retail environment, which has a relatively weak collaborative relationship with the GP sector in Ireland.^40^ This was partially reflected in our analysis as the different correlation between some covariates and telemedicine outcomes (Table S5). The coefficients for ICT access for GP and pharmacists were similar, while the insignificant correlation between hospital care may be explained by the different content delivered by primary care specialists and hospital doctors. For example, hospital care may have initially been carried out in the hospital setting, and then followed up by scheduled telemonitoring, teleconsultation, and primary care when hospital capacities were relatively more constrained during the pandemic.^41^ Remote healthcare provision from the hospital setting may have been initiated and driven by healthcare specialists as part of a specific medical treatment. On the other hand, appointments with GPs and pharmacists may have been greater driven by patients in relation to the management of chronic diseases or other medical consultations.

Further analysis revealed that the results were not driven by more socio-economically advantaged groups, and the association was more prominent among a younger group (those aged under 70 years) and those located in non-Dublin urban areas. The modeling estimates showed that people with more chronic conditions, poorer mental health, and private health insurance had higher odds of using telemedicine in the period of study. Furthermore, there was no clear pattern in the uptake of telemedicine by different GP accessibility, suggesting little evidence of a substitutive or complementary relationship between telemedicine and in-person health care services.

In relation to the framework of TAM,^21^^,^^24^ our results suggest a greater influence from the infrastructure factor (home internet access) than other factors related to people’s perceived benefits and ease of using technological tools which could be partially reflected by general ICT activity level and GP accessibility.

The study has several limitations which must be acknowledged. First, the measure of telemedicine use was self-reported and lacks further detailed information such as the service provider (private or public health service), or the specific nature of the telemedicine appointment. Also, the measure did not differentiate between telephone and video consultation. Since there was likely to be a higher proportion of telephone-based consultations among the older adults,^6^^,^^42^ our interpretation of the association between ICT factors such as availability of high-speed broadband on telemedicine use might be limited in some ways when video consultation was relatively more constrained by ICT infrastructure. Second, regarding the scope of time, we primarily concentrated on telemedicine usage within the first year of the pandemic, making it difficult to extrapolate our findings to a more generalized setting. The timing of the data available, related especially to healthcare utilization during the pandemic period, which limited an analysis of the general trend of increasing telemedicine usage. However, given that the main purpose of this study was to examine ICT-related influences, the findings appeared to be less affected by pandemic-specific factors such as healthcare delay and people’s concern over COVID, which was also consistent with the US study by Alexander et al. In addition, essential activities such as exercise and healthcare were allowed at the local level,^43^ and the closure of non-essential outlets and services at the time was also less influential for our estimates as the internet access variable relates to the home. Regional COVID burden also had little impact on our results as the exposure and infection of COVID-19 were similar across regions, and most of the surveyed respondents reported negative test results at the time of the interview.^39^ In relation to the lack of an effect of people’s concern about the pandemic on our estimated relationship between ICT factors and telemedicine, this could be due to ambiguity in the measure employed, which enquired about the general sense of the overall uncertain impacts of the pandemic on broader issues such as the economy and population.^39^ Third, our analysis did not reveal a causal relationship although our results reflected a longitudinal association and may be less threatened by unobservables.

In contrast to many other European countries, Ireland does not have universal coverage of public health insurance for primary and community health services, and out-of-pocket medical spending is relatively high.^44^ Research suggests the role of geographical factors in accessing general practice care and community care.^32^^,^^33^ In addition to limited publicly funded pilot projects, the majority of telemedicine is privately provided and financed, and in recent years private providers are increasingly offering access to remote consultations.^45^ Our results suggest an important role of access to and frequent engagement with the Internet in encouraging telemedicine usage among older people.

Future research is needed to examine the degree of adoption of telemedicine post the pandemic to assess whether telemedicine was only acceptable as a temporary stopgap in times of crisis, or whether it will become an integrated part of healthcare delivery on a longer-term basis. Moreover, studies further linking administrative/GP practice data would better speak to potential changing preferences toward remote healthcare. Future studies in this area are also expected to provide a greater understanding of the finance and cost implications of remote healthcare delivery, for instance, investigations of whether the use of telemedicine gives rise to cost savings for patients and the healthcare system.

## Supplementary Material

ocad165_Supplementary_Data

## Data: The Irish Longitudinal Study on Ageing (TILDA)

The publicly accessible dataset files are hosted by the Irish Social Science Data Archive based at University College Dublin, and the Interuniversity Consortium for Political and Social Research (ICPSR) based at the University of Michigan. Researchers wishing to access the data must complete a request form, available on either the ISSDA* or ICPSR website.
