# Supplementary material for: Use of information communication technologies by older people and telemedicine adoption during COVID-19: a longitudinal study
Source: J Am Med Inform Assoc. 2023 Aug 12;30(12):2012–20. doi: 10.1093/jamia/ocad165 (PMC10654849; doi:10.1093/jamia/ocad165)
Supplement: ocad165_Supplementary_Data [file ocad165_supplementary_data.docx]

# **Supplementary Files**

Supplementary material A: **Survey questions on ICT usage**

1. Do you have access to the Internet?
2. I can access it at home
3. I can access it elsewhere (friend/relative’s house, library, community centre etc.)
4. I have no access to the internet
5. On which of the following devices do you access the internet?
6. Desktop computer
7. Laptop computer
8. Tablet computer (e.g., iPad, Samsung Galaxy Tab)
9. Smartphone (e.g., iPhone, Blackberry)
10. TV (e.g., games console or set top box)
11. Others
12. For which of the following activities did you use the internet in the last 3 months?
13. Sending/receiving e-mails
14. Telephoning or using video calls (via webcam) over the internet to stay in contact with family or friends (e.g., skype)
15. Searching for information for learning, research, fact finding
16. Financial transactions (e.g., online shopping, buying or selling goods or services, banking, paying bills, booking flights)
17. Using social networking sites (e.g., Facebook, Twitter, Myspace)
18. News/newspaper/blog website
19. Gaming/Apps
20. Others

Supplementary material B: **Data explanations on GP accessibility**

1. Road network distance (kilometres) from TILDA respondent’s residence (postcode) to the nearest GP (postcode). The road network distance in kilometres was derived from OpenStreetMap which is a superior measure of geographical proximity than straight-line Euclidian distances since it reflects actual distances along roads. This variable provides an indication of geographical proximity. A lower value represents a shorter distance to a GP for respondents.
2. The number of residential addresses potentially served by the nearest GP. Data on all residential addresses in Ireland from the An Post Geodirectory were used to estimate the number of addresses to which each GP practice is the nearest, again using network distance. Each address in the country was assigned to its nearest GP. This indicator acts as a proxy for the workload/congestion/capacity of the local GP. This variable is novel in terms of the existing literature on accessibility. A lower value represents a lower ‘workload’ of the local GP for respondents.
3. The number of GPs within walking distance (1.6km radius) of a respondent’s residence. This variable provides an indication of the availability/density/choice of primary care providers available to respondents in their locality. The 1.6km buffer, based on Euclidean distance, equates to a twenty-minute walk and has been used extensively in other studies of walking distance (Sturm and Cohen, 2014). A lower value represents a lower availability of the local GP for respondents.

In our analysis, all the variables were used in quantiles to protect respondent anonymity and to take account of the skewness of the original variables. Within three general regions (Dublin, other cities/towns, rural area), three quantile groups were constructed to represent the 0-33% (lowest 33%), 33-66%, top 33% of the original variables. For the number of GPs in the area, we reversed the value for the lowest and the top group to keep a consistent indication as the other two variables (distance and number of residential addresses), that is, the higher quantile value all represents a lower GP accessibility.

Reference: Sturm R, Cohen D. Proximity to urban parks and mental health. The journal of mental health policy and economics. 2014 Mar;17(1):19.

Table S1 Summary statistics of telemedicine and internet variables

|  | Mean | Std. Dev | Mean | Mean |
| --- | --- | --- | --- | --- |
|  | All | | Male | Female |
|  | (N=2,607) | | (N=1,115) | (N=1,490) |
| **Telemedicine** |  |  |  |  |
| Any health service | 0.525 | 0.499 | 0.538 | 0.515 |
| General practitioner (GP) | 0.361 | 0.480 | 0.374 | 0.352 |
| Pharmacist | 0.255 | 0.436 | 0.248 | 0.261 |
| Hospital doctor | 0.114 | 0.318 | 0.116 | 0.113 |
| **Internet access and devices** |  |  |  |  |
| Internet access | 0.864 | 0.343 | 0.860 | 0.867 |
| High-speed broadband | 0.717 | 0.451 | 0.715 | 0.718 |
| Personal computer | 0.705 | 0.456 | 0.734 | 0.684 |
| Tablet/smartphone | 0.757 | 0.429 | 0.727 | 0.779 |
| Other mobile device/TV | 0.392 | 0.488 | 0.390 | 0.394 |
| **Internet use frequency** |  |  |  |  |
| Daily user | 0.633 | 0.482 | 0.611 | 0.650 |
| Weekly user | 0.140 | 0.347 | 0.143 | 0.138 |
| Monthly user | 0.040 | 0.197 | 0.046 | 0.036 |
| Every three months | 0.019 | 0.136 | 0.023 | 0.016 |
| Non-user | 0.168 | 0.374 | 0.178 | 0.160 |
| **Recent internet activities** |  |  |  |  |
| Email | 0.670 | 0.470 | 0.646 | 0.688 |
| Telephone/video | 0.390 | 0.488 | 0.356 | 0.416 |
| Search for information | 0.727 | 0.446 | 0.714 | 0.736 |
| Finance | 0.555 | 0.497 | 0.548 | 0.560 |
| Social media | 0.345 | 0.476 | 0.263 | 0.407 |
| News/newspapers/blogs | 0.516 | 0.500 | 0.532 | 0.504 |
| Gaming/apps | 0.152 | 0.359 | 0.130 | 0.168 |

Table S2 Model specification checks

| Specification: | (1) | (2) | (3) | (4) | (5) |
| --- | --- | --- | --- | --- | --- |
| Panel A: Any health service | | | | | |
| Internet access | 1.436** | 1.529** | 1.587*** | 1.604*** | 1.608*** |
|  | (0.219) | (0.255) | (0.264) | (0.281) | (0.269) |
| N | 2,607 | 2,607 | 2,607 | 2,595 | 2,567 |
| Highspeed broadband | 1.038 | 0.973 | 0.991 | 1.001 | 0.990 |
|  | (0.139) | (0.132) | (0.134) | (0.134) | (0.143) |
| N | 2,571 | 2,571 | 2,571 | 2,559 | 2,567 |
| Panel B: GP |  |  |  |  |  |
| Internet access | 1.451** | 1.592*** | 1.622*** | 1.689*** | 1.657*** |
|  | (0.232) | (0.277) | (0.279) | (0.302) | (0.290) |
| N | 2,607 | 2,607 | 2,607 | 2,595 | 2,567 |
| Highspeed broadband | 1.143 | 1.094 | 1.118 | 1.116 | 1.103 |
|  | (0.165) | (0.157) | (0.161) | (0.160) | (0.170) |
| N | 2,571 | 2,571 | 2,571 | 2,559 | 2,567 |
| Panel C: Pharmacist |  |  |  |  |  |
| Internet access | 1.441** | 1.607** | 1.676*** | 1.676*** | 1.654*** |
|  | (0.261) | (0.304) | (0.316) | (0.326) | (0.311) |
| N | 2,607 | 2,607 | 2,607 | 2,595 | 2,567 |
| Highspeed broadband | 0.888 | 0.836 | 0.846 | 0.848 | 0.836 |
|  | (0.142) | (0.134) | (0.135) | (0.137) | (0.146) |
| N | 2,571 | 2,571 | 2,571 | 2,559 | 2,567 |
| Panel D: Hospital doctor |  |  |  |  |  |
| Internet access | 0.978 | 1.160 | 1.192 | 1.165 | 1.222 |
|  | (0.236) | (0.294) | (0.298) | (0.308) | (0.310) |
| N | 2,607 | 2,607 | 2,607 | 2,595 | 2,567 |
| Highspeed broadband | 0.793 | 0.728 | 0.767 | 0.753 | 0.768 |
|  | (0.184) | (0.174) | (0.180) | (0.179) | (0.181) |
| N | 2,571 | 2,571 | 2,571 | 2,559 | 2,567 |
| Included Covariates |  |  |  |  |  |
| Demographic | YES | YES | YES | YES | YES |
| Socioeconomic | NO | YES | YES | YES | YES |
| Health | NO | NO | YES | YES | YES |
| Healthcare utilisation | NO | NO | NO | YES | NO |
| GP accessibility | NO | NO | NO | NO | YES |

*Note*: * p<0.1; **p<0.05; ***p<0.01. Standard errors in parenthesis were clustered at the household level. Odds ratios reported. Estimates were adjusted by survey weights. Demographic variables included age, age squared, gender, residence location, marital status, whether born in Ireland, household size and early childhood conditions. Socioeconomic characteristics included education, current labour market status, quartiles of household gross assets, house ownership, and healthcare entitlements. Health variables included self-rated health status, chronic conditions, disability, and self-rated mental health. Healthcare variables included the number of GP visits, hospital outpatient and inpatient visits. GP accessibility variables include the network distance to GP, the number of GPs within a walking distance, and the total number of GPs in a catchment area. All control variables were taken from wave 5.

Table S3 Robustness checks: estimations in alternative samples

|  | (1) | (2) | (3) | (4) | (5) |  |
| --- | --- | --- | --- | --- | --- | --- |
| Subsamples: | Baseline | Has delay | No delay | GP visit | COVID concern |  |
| Panel A: Internet access at wave 5 | | |  |  |  |  |
| Any health service | 1.587*** | 1.012 | 1.709*** | 1.508** | 1.513* |  |
|  | (0.264) | (0.355) | (0.336) | (0.261) | (0.337) |  |
| GP | 1.622*** | 1.391 | 1.667** | 1.585** | 1.601** |  |
|  | (0.279) | (0.433) | (0.343) | (0.288) | (0.368) |  |
| Pharmacist | 1.676*** | 1.861* | 1.552** | 1.776*** | 1.928*** |  |
|  | (0.316) | (0.668) | (0.339) | (0.362) | (0.466) |  |
| Hospital doctor | 1.192 | 1.113 | 1.067 | 0.940 | 1.460 |  |
|  | (0.298) | (0.490) | (0.338) | (0.262) | (0.479) |  |
| N | 2,607 | 756 | 1,851 | 2,378 | 1,756 |  |
| N(clusters) | 2,045 | 704 | 1,546 | 1,903 | 1,462 |  |
| Panel B: High-speed broadband availability | | |  |  |  |  |
| Any health service | 0.991 | 0.440*** | 1.325* | 1.017 | 0.781 |  |
|  | (0.134) | (0.117) | (0.215) | (0.145) | (0.135) |  |
| GP | 1.118 | 0.764 | 1.370* | 1.111 | 0.897 |  |
|  | (0.161) | (0.196) | (0.242) | (0.169) | (0.162) |  |
| Pharmacist | 0.846 | 0.536** | 0.958 | 0.841 | 0.685* |  |
|  | (0.135) | (0.156) | (0.178) | (0.143) | (0.137) |  |
| Hospital doctor | 0.767 | 0.443** | 1.033 | 0.676 | 0.630 |  |
|  | (0.180) | (0.167) | (0.325) | (0.170) | (0.181) |  |
| N | 2,571 | 753 | 1,818 | 2,345 | 1,730 |  |

*Note*: p<0.1; ** p<0.05; *** p<0.01. Standard errors in parentheses were clustered at the household level. Odds ratios reported. (2)(3) divided the sample by whether has health service or care delay during the COVID pandemic. (4) excluded the people with the top 10% number of GP visits. (5) excluded people reporting extreme concerns over COVID.

Table S4 Heterogeneities by region and education level

|  | (1) | (2) | (3) | (4) | (5) | (6) |
| --- | --- | --- | --- | --- | --- | --- |
|  | Residence area | | | Education level | | |
|  | Dublin | Other City | Rural areas | Primary | Secondary | Third/higher |
| Panel A: Internet access | | | | | | |
| Any health services | 1.057 | 3.183*** | 1.113 | 1.267 | 1.996*** | 1.350 |
|  | (0.451) | (1.006) | (0.246) | (0.319) | (0.460) | (0.557) |
| GP | 1.505 | 3.310*** | 1.204 | 1.432 | 2.120*** | 1.368 |
|  | (0.623) | (1.026) | (0.288) | (0.381) | (0.526) | (0.561) |
| Pharmacist | 0.865 | 3.524*** | 0.934 | 1.597 | 1.355 | 0.913 |
|  | (0.382) | (1.279) | (0.247) | (0.457) | (0.352) | (0.362) |
| Hospital doctor | 0.649 | 1.837 | 1.214 | 1.073 | 1.970* | 2.315 |
|  | (0.372) | (0.839) | (0.429) | (0.394) | (0.728) | (1.597) |
| Mean of ICT | 0.928 | 0.840 | 0.840 | 0.600 | 0.864 | 0.959 |
| Mean of service use (any) | 0.562 | 0.535 | 0.497 | 0.523 | 0.526 | 0.525 |
| N | 694 | 727 | 1,186 | 417 | 1,033 | 1,157 |
| N(clusters) | 545 | 588 | 912 | 392 | 922 | 1,013 |
| Panel B: High-speed broadband availability | | | | | | |
| Any health services | 1.835 | 0.516* | 1.108 | 0.929 | 1.009 | 0.845 |
|  | (1.147) | (0.179) | (0.173) | (0.278) | (0.187) | (0.182) |
| GP | 2.772 | 0.682 | 1.286 | 0.816 | 1.296 | 1.159 |
|  | (2.339) | (0.233) | (0.211) | (0.276) | (0.251) | (0.276) |
| Pharmacist | 2.160 | 0.570* | 0.858 | 1.582 | 0.650** | 0.548** |
|  | (1.849) | (0.189) | (0.164) | (0.583) | (0.138) | (0.133) |
| Hospital doctor | 1.222 | 0.212*** | 1.284 | 0.949 | 0.719 | 0.612 |
|  | (1.355) | (0.089) | (0.333) | (0.444) | (0.232) | (0.226) |
| Mean of ICT | 0.972 | 0.892 | 0.460 | 0.663 | 0.712 | 0.741 |
| Mean of service use (any) | 0.560 | 0.538 | 0.501 | 0.525 | 0.527 | 0.528 |
| N | 684 | 719 | 1,168 | 413 | 1,021 | 1,137 |

*Note*: *p<0.1; ** p<0.05; *** p<0.01. Standard errors in parenthesis were clustered at household level. Odds ratios reported. Estimates were adjusted by survey weights.

Table S5: Multivariate estimation results: Association between other covariates and telemedicine, odds ratios

| Telemedicine type: | Any | GP | Pharmacist | Hospital doctor |
| --- | --- | --- | --- | --- |
|  | (1) | (2) | (3) | (4) |
| Internet Access | 1.587*** | 1.622*** | 1.676*** | 1.192 |
|  | (0.264) | (0.279) | (0.316) | (0.298) |
| Age | 0.854 | 0.789** | 1.139 | 0.963 |
|  | (0.092) | (0.084) | (0.136) | (0.148) |
| Age^2/100 | 1.105 | 1.171** | 0.916 | 1.018 |
|  | (0.083) | (0.087) | (0.075) | (0.109) |
| Male | 1.093 | 1.050 | 1.096 | 1.172 |
|  | (0.120) | (0.119) | (0.139) | (0.200) |
| Education: secondary | 1.153 | 1.099 | 0.890 | 0.982 |
| (ref: primary/none) | (0.167) | (0.168) | (0.152) | (0.209) |
| Education: third/higher | 1.242 | 1.026 | 0.905 | 1.100 |
|  | (0.201) | (0.177) | (0.178) | (0.259) |
| Other cities | 0.831 | 0.952 | 1.436** | 0.742 |
| (ref: Dublin) | (0.125) | (0.150) | (0.261) | (0.176) |
| Rural areas | 0.739** | 0.787 | 1.231 | 0.595** |
|  | (0.103) | (0.118) | (0.215) | (0.128) |
| Never married | 0.862 | 0.693 | 0.832 | 0.864 |
| Marital status (ref: married) | (0.190) | (0.162) | (0.231) | (0.278) |
| Separated/divorced | 1.254 | 1.178 | 1.147 | 1.276 |
|  | (0.290) | (0.272) | (0.297) | (0.429) |
| Widowed | 0.826 | 0.661** | 1.032 | 0.780 |
|  | (0.141) | (0.120) | (0.207) | (0.217) |
| Household size | 0.955 | 0.921 | 1.023 | 1.076 |
|  | (0.056) | (0.060) | (0.076) | (0.100) |
| Retired | 1.353** | 1.272 | 1.591*** | 1.169 |
| (ref: employed/self-employed) | (0.198) | (0.199) | (0.283) | (0.261) |
| Unemployed/homemaker/sick/other | 1.162 | 1.211 | 2.248*** | 1.485 |
|  | (0.215) | (0.230) | (0.461) | (0.414) |
| Never worked | 1.542* | 1.354 | 2.609*** | 1.090 |
|  | (0.398) | (0.365) | (0.752) | (0.495) |
| Born in Ireland | 1.556** | 1.364* | 0.951 | 1.296 |
|  | (0.278) | (0.257) | (0.193) | (0.333) |
| Childhood SES: average | 0.737* | 0.790 | 0.790 | 1.336 |
| (ref: excellent) | (0.122) | (0.134) | (0.148) | (0.317) |
| Childhood SES: poor | 0.823 | 0.888 | 0.880 | 0.768 |
|  | (0.167) | (0.185) | (0.200) | (0.235) |
| Childhood health: very good | 1.212 | 1.139 | 1.291* | 1.153 |
| (ref: excellent) | (0.152) | (0.145) | (0.182) | (0.227) |
| Childhood health: good | 0.908 | 0.864 | 0.950 | 1.323 |
|  | (0.157) | (0.160) | (0.196) | (0.350) |
| Childhood health: fair/poor | 1.542* | 1.149 | 1.928*** | 1.471 |
|  | (0.360) | (0.255) | (0.453) | (0.422) |
| Household gross assets : 20% - 40% | 0.785 | 0.802 | 0.924 | 0.654 |
| (ref: lowest 20%) | (0.183) | (0.187) | (0.234) | (0.234) |
| Household gross assets: 40%- 60% | 1.385 | 1.197 | 1.205 | 1.160 |
|  | (0.324) | (0.281) | (0.305) | (0.383) |
| Household gross assets: 60%-80% | 0.931 | 0.649* | 1.327 | 0.857 |
|  | (0.225) | (0.159) | (0.359) | (0.304) |
| Household gross assets: top 20% | 1.127 | 0.904 | 1.249 | 0.891 |
|  | (0.285) | (0.230) | (0.350) | (0.334) |
| Missing asset information | 0.944 | 0.861 | 0.941 | 0.785 |
|  | (0.210) | (0.193) | (0.229) | (0.266) |
| House ownership | 0.947 | 0.989 | 0.586* | 0.802 |
|  | (0.246) | (0.250) | (0.162) | (0.263) |
| Dural insurance | 0.951 | 1.063 | 1.158 | 0.955 |
| (ref: medical/gp card) | (0.148) | (0.174) | (0.208) | (0.219) |
| Only private insurance | 0.522*** | 0.592*** | 0.863 | 0.484*** |
|  | (0.093) | (0.108) | (0.178) | (0.130) |
| Uncovered | 0.657* | 0.746 | 0.947 | 0.622 |
|  | (0.148) | (0.175) | (0.278) | (0.231) |
| Self-rated health: very good | 1.250 | 1.206 | 1.014 | 0.893 |
| (ref: excellent) | (0.219) | (0.231) | (0.218) | (0.263) |
| Self-rated health: good | 1.281 | 1.147 | 0.950 | 0.818 |
|  | (0.247) | (0.239) | (0.229) | (0.251) |
| Self-rated health: fair/poor | 1.650* | 1.217 | 0.968 | 1.852* |
|  | (0.432) | (0.308) | (0.278) | (0.674) |
| 1-2 chronic conditions | 1.403** | 1.385** | 1.172 | 1.615 |
| (ref: no chronic disease) | (0.212) | (0.229) | (0.224) | (0.493) |
| >=3 chronic conditions | 1.871*** | 1.766*** | 1.581** | 2.231** |
|  | (0.323) | (0.319) | (0.330) | (0.748) |
| IADL only | 0.831 | 0.803 | 0.971 | 0.443 |
| (ref: no disability) | (0.338) | (0.319) | (0.393) | (0.255) |
| Any ADL | 1.025 | 0.924 | 1.169 | 0.863 |
|  | (0.232) | (0.196) | (0.273) | (0.273) |
| Mental health: very good | 1.171 | 1.188 | 1.379* | 0.962 |
| (ref: excellent) | (0.193) | (0.208) | (0.256) | (0.241) |
| Mental health: good | 1.155 | 1.251 | 1.701** | 1.218 |
|  | (0.207) | (0.235) | (0.357) | (0.319) |
| Mental health: fair/poor | 1.732** | 1.703** | 1.795** | 1.729 |
|  | (0.473) | (0.435) | (0.511) | (0.602) |
| N | 2,607 | 2,607 | 2,607 | 2,607 |

*Note:* * p<0.1; **p<0.05; ***p<0.01. Standard errors in parenthesis were clustered at the household level. Odds ratios reported. Estimates were adjusted by survey weights.
